# Comparison of MicroRNA Transcriptomes Reveals the Association between MiR-148a-3p Expression and Rumen Development in Goats

**DOI:** 10.3390/ani10111951

**Published:** 2020-10-23

**Authors:** Tao Zhong, Cheng Wang, Jiangtao Hu, Xiaoyong Chen, Lili Niu, Siyuan Zhan, Linjie Wang, Jiazhong Guo, Jiaxue Cao, Li Li, Hongping Zhang

**Affiliations:** 1 Farm Animal Genetic Resources Exploration and Innovation Key Laboratory of Sichuan Province, College of Animal Science and Technology, Sichuan Agricultural University, Chengdu 611130, China; zhongtao@sicau.edu.cn (T.Z.); wxx79595@163.com (C.W.); jiusi1990@163.com (J.H.); siyuanzhan@sicau.edu.cn (S.Z.); wanglinjie@sicau.edu.cn (L.W.); jiazhong.guo@sicau.edu.cn (J.G.); jiaxuecao@sicau.edu.cn (J.C.); lily@sicau.edu.cn (L.L.); zhp@sicau.edu.cn (H.Z.); 2 College of Animal Science and Technology, Hebei Agricultural University, Baoding 071001, China; chenxiaoyong@hebau.edu.cn

**Keywords:** goat, rumen, RNA sequencing, microRNA, proliferation

## Abstract

**Simple Summary:**

In ruminants, the rumen epithelium plays an important role in nutrient absorption, metabolism and transport. MicroRNAs (miRNAs) have been reported to regulate the proliferation of diverse epithelial cells. In this study, we profiled the miRNA transcriptomes of goat rumens at four development stages and screened for candidate miRNAs related to rumen development. MiR-148a-3p was found to be highly expressed in the rumen tissues and induced the proliferation of GES-1 cells by targeting *QKI*. Our findings provide some insights into the functional roles of miRNAs in rumen growth and functional development in ruminants.

**Abstract:**

The rumen is an important digestive organ of ruminants. From the fetal to adult stage, the morphology, structure and function of the rumen change significantly. However, the knowledge of the intrinsic genetic regulation of these changes is still limited. We previously reported a genome-wide expression profile of miRNAs in pre-natal goat rumens. In this study, we combined and analyzed the transcriptomes of rumen miRNAs during pre-natal (E60 and E135) and post-natal (D30 and D150) stages. A total of 66 differentially expressed miRNAs (DEMs) were identified in the rumen tissues from D30 and D150 goats. Of these, 17 DEMs were consistently highly expressed in the rumens at the pre-weaning stages (E60, E135 and D30), while down-regulated at D150. Noteworthy, annotation analysis revealed that the target genes regulated by the DEMs were mainly enriched in MAPK signaling pathway, Jak-STAT signaling pathway and Ras signaling pathway. Interestingly, the expression of miR-148a-3p was significantly high in the embryonic stage and down-regulated at D150. The potential binding sites of miR-148a-3p in the 3′-UTR of *QKI* were predicted by the TargetScan and verified by the dual luciferase report assay. The co-localization of miR-148a-3p and *QKI* through in situ hybridization was observed in the rumen tissues but not in the intestinal tracts. Moreover, the expression of miR-148a-3p in the epithelium was significantly higher than that in the other layers of the rumen, suggesting that miR-148a-3p is involved in the development of the rumen epithelial cells by targeting *QKI*. Subsequently, miR-148a-3p inhibitor was found to induce the proliferation of GES-1 cells. Taken together, our study identified DEMs involved in the development of the rumen and provides insights into the regulation mechanism of rumen development in goats.

## 1. Introduction

The rumen is the largest compartment of stomach in adult ruminants, which plays an important role in the digestion and absorption of nutrients. The development of the rumen in goats includes the development of tissue’s morphology and metabolic function. The rumen wall is thin and slightly transparent, and it is divided into five layers including the epithelial layer, lamina propria, submucosa, muscular layer and serous layer [1]. In mature ruminants the rumen epithelium is involved in many important physiological functions, including absorption, transportation, volatile fatty acid (VFA) metabolism, and protection [2]. In addition, a large number of microorganisms in rumen also play a significant role in the processes of digestion and absorption [3]. Up to now, the effects of dietary composition [4] and weaning age [5] on rumen development have been widely investigated, while the roles of microRNAs in goat rumen development have been rarely described.

MicroRNA (miRNA), first identified in C. elegans, is a class of small non-coding RNA [6]. They mainly regulate the expression of their target gene/genes by binding to the seed region of the 3′-untranslated region (3′-UTR) of the target gene/gens [7]. MiRNAs can regulate gene expression and participate in the regulation of various biological processes [8]. In sheep and goats, some miRNAs have been shown to play crucial roles in skeletal myogenesis [9,10,11], fat deposition [12], adipogenic differentiation [13], lipid metabolism in mammary gland [14,15], testes development [16]. To date, it has been shown that several miRNAs are related with rumen development and miR-148a-3p is one of the most abundant miRNAs in goat rumen during embryonic stage [17]. Furthermore, previous studies have revealed that miR-148a-3p regulates the proliferation and apoptosis of skeletal muscle cells by post-transcriptional regulation of target gene in cattle [18] and chicken [19]. Quaking (QKI) is an RNA binding protein and found to be vital to mammalian embryonic development. *QKI* could regulate glial cell function by regulating the expression of specific miRNAs [20]. Meanwhile, miR-214 inhibited angiogenesis via direct targeting of *QKI* and reduced the release of pro-angiogenic growth factor [21]. In additional, it has recently been proposed that miR-148a-3p is involved in apoptosis and/or cell adhesion of osteosarcoma cell lines by potentially inhibiting *QKI* [22].

In our previous study, the expression profiles of the rumen tissue miRNAs were explored in two embryonic stages (E60 and E135) [17]. In the current study, we investigated differentially expressed miRNAs (DEMs) identified between pre-weaning (D30) and post-weaning stage (D150), and jointly analyzed the RNA sequencing data from our previous study. Furthermore, we identified miR-148a-3p regulated the expression of *QKI* by directly targeting 3′-UTR of *QKI*. Inhibition of the expression of miR-148a-3p could significantly improve the proliferation capacity of GES-1 cells, suggesting that miR-148a-3p may be involved in the process of rumen development in goats.

## 2. Materials and Methods

### 2.1. Experimental Animals and Rumen Tissue Collection

The goats used in this experiment were provided by the Jianzhou Da’er Goat Breeding Center (Sichuan, China). Twelve rumen tissues were collected from goats at four different periods (three samples at each stage; 60 and 135 days of gestation, 30 and 150 days after birth, represented as E60, E135, D30, and D150, respectively). Goats were reared according to the local feeding standard (DB51/T 1750-2014). Goat kids were weaned at D60. The pre-natal samples were collected by cesarean section [17], while the post-natal rumens were dissected and frozen in liquid nitrogen immediately after slaughter, and then stored at −80 °C. All experiments involving animals were conducted according to the Regulations for the Administration of Affairs Concerning Experimental Animals (Ministry of Science and Technology, China, revised in June 2004).

### 2.2. RNA Isolation, Library Construction, and Sequencing

Total RNA was extracted from rumen tissues using TRIzol (Invitrogen, Carlsbad, CA, USA), according to the manufacturer’s instructions. The purity, concentration, and quality of RNA were determined by a Nanodrop 2000 spectrophotometer, Qubit 3.0 Fluorometer, and Agilent 2100 Bioanalyzer, respectively. Small RNA libraries were constructed with 1.5 µg of total RNA using the TruSeq small RNA Sample Pre Kit (Illumina, San Diego, CA, USA). Finally, twelve libraries were sequenced on Illumina Hiseq 2500 platform with single-end reads. Goat rumen RNA-Seq data are available at the SRA database (PRJNA596079).

### 2.3. miRNAs Data Analyses and qPCR Validation

Two datasets including the newly obtained raw data (D30 and D150) and our previous data (E60 and E135) [17] were jointly analyzed in the present study. The procedures of miRNA identification and expression were performed according to the methods [17,23]. Differential miRNA expression was analyzed using the DESeq R package. DEMs were identified using the criteria of |log2 (fold change)| ≥ 1 and *p*-value ≤ 0.01. The miRnada [24] and RNAhybrid [25] were used to predict the target genes of DEMs. Blast software was used to compare the predicted target gene sequences with Gene Ontology (GO) and Kyoto Encyclopedia of Genes and Genomes (KEGG) databases to obtain the annotation information of target genes. To verify the high-throughput sequencing data, we randomly selected 8 miRNAs from the identified DEMs by qRT-PCR. Total RNA (5 μg) was reverse transcribed into cDNA using the Mir-XTM miRNA First-Strand Synthesis Kit (TaKaRa, Dalian, China). The primer sequences for qRT-PCR are listed in Supplementary Table S1. qRT-PCR was performed using the SYBR PrimeScript miRNA RT-PCR Kit (Takara) on a CFX Connect Real-Time PCR Detection System (Bio-Rad Laboratories, Singapore). Briefly, a volume of 10 µL including 5 µL SYBR Green Real Time PCR Master Mix (TaKaRa), 0.2 µL (10 µM) each of forward and reverse primer, 3.8 µL RNase-Free ddH_2_O, and 50 ng cDNA. qPCR reactions were performed using the following condition: 95.0 °C for 30 s, and 40 cycles of 95.0 °C for 5 s, Tm for 30 s. U6 was used as a reference gene. The expression level of miRNA was calculated with the 2^−ΔΔCT^ method [26]. All samples were run in triplicate.

### 2.4. Expression Vector Construction

The putative binding sites of miR-148a-3p in the 3′-UTR of *QKI* were predicted the TargetScan website (www.targetscan.org). The wild-type (wt) 3′-UTR of *QKI* was amplified by PCR. Sequences of the primers used in vector construction assays are listed in Supplementary Table S1. PCR amplification system contained a 30 µL volume including 2× Master Mix Taq, forward and reverse primers 2 µL (10 µM), cDNA 50 ng and ddH_2_O 12 µL. The reaction cycler conditions: 95.0 °C for 5 min; 95.0 °C for 30 s, Tm for 30 s, 72 °C for 30 s, 35 cycles; 72 °C 5 min. The amplified product and the psiCHECK-2 vector (Promega, Madison, WI, USA) were digested. The digestion system was placed at 37 °C for 2 h. The digestion products were recovered and incubated at 16 °C overnight using T4 ligase. The DH5α (TIANGEN, Beijing, China) competent cells (50 μL) were added to the ligation, culled from a single colony, identified and verified by sequencing. The psi-CHECK2 plasmid containing the mutant type (mut) 3′-UTR of *QKI* was obtained from GeneCreate Biological Engineering (Wuhan, China).

### 2.5. Cell Culture, Transfection, and Expression Detection

HEK293T cells were used to detect luciferase activity, and human gastric cancer cell line GES-1 (BNCC, Beijing, China) cells were used to detect the effect of miR-148a-3p on cell proliferation. Cells were maintained in DMEM supplied with 10% FBS at a 37 °C incubator containing 5% CO_2_. LipofectamineTM 2000 (Invitrogen, Carlsbad, CA, USA) was used for cell transfection following the manufacturer’s procedure. GES-1 cells were transfected with miR-148a-3p mimics, miR-148a-3p inhibitor, miR-148a-3p-NC, *QKI* siRNA, or *QKI*-NC at the concentration of 50 nM, respectively. miR-148a-3p mimics and mimic control were chemically synthesized by Ribo Bio Co. Ltd. (Guangzhou, China). The expression levels of miR-148a-3p and *QKI* isoforms in each group were detected by qPCR at 48 h post-transfection. *QKI5*, *QKI6*, and *QKI7* primers were designed in their corresponding specific sequence positions (Supplementary Figure S1).

### 2.6. Luciferase Reporter Assay

For luciferase reporter assay, HEK293T cells were co-transfected with miR-148a-3p mimics (or miR-148a-3p NC) and wt-QKI-3′-UTR plasmids (or mut-QKI-3′-UTR) using Lipofectamine 2000 (Invitrogen, Carlsbad, CA, USA). After transfection 48 h, the luciferase activities were measured on a GloMax^®^ 96 Microplate Luminometer (Promega) by the TransDetect Double-Luciferase Reporter Assay Kit (TaKaRa).

### 2.7. Cell Proliferation Assays

The GES-1 cells was used to study cell proliferation by the Cell Counting kit-8 (Dojindo, Shanghai, China). The GES-1 cells were seeded into 96-well plates at a density of 1 × 10^4^ cells per well determined by the Corning Cell Counter (Corning Incorporated, Corning, NY, USA). After transfection of the miR-148a-3p mimics or the miR-148a-3p-NC, the cells were cultured for 4, 24, 48 and 72 h. Then, the absorbance of cells was measured with a wavelength of 450 nm using a microplate reader (Analytik Jena AG, Jena, Germany).

### 2.8. Dual-Color Fluorescence In Situ Hybridization

Briefly, tissue sections were deparaffinized, dehydrated, and incubated in room temperature for 15 min, and digested in pepsin solution for 3–30 min at 37 °C. Denaturation, hybridization, and post-hybridization washings were carried out by following the manufacturer’s instructions (Wuhan GeneCreate Biological Engineering Co., Ltd., China). After DAPI (10 μg/mL) staining for 10 min, washing with 0.5 × SSC for 15 min and washing with 0.2 × SSC for 15 min at 37 °C were carried out. MiR-148a-3p was labeled with red, while *QKI* was labeled with green. Images were captured by an Olympus BX51T fluorescence microscope (Olympus, Tokyo, Japan) and analyzed by the Image-Pro Plus software (Media Cybernetics, Rockville, MA, USA).

### 2.9. Statistical Analyses

Statistical analyses were conducted using SPSS 20.0 software (IBM, Armonk, NY, USA). Data were analyzed by one-way analysis of variance (ANOVA). The differences between the groups were analyzed using a Student’s t-test and multiple comparison tests. The differences between the means were considered statistically significant when *p* < 0.05.

## 3. Results

### 3.1. miRNAs Expression Profile and DEMs in Goat Rumens

In this study, an average of 18.59 million single-end clean reads were generated, and approximately 50.6% mapped to the goat reference genome ARS1 (Supplementary Table S2). A total of 1488 miRNAs were identified in the rumen samples, including 431 known miRNAs and 1057 novel miRNAs (Supplementary Table S3). The top-10 miRNAs are listed in Table 1. Seven miRNAs (miR-143-3p, let-7i-5p, miR-148a-3p, let-7f-5p, miR-21-5p, miR-26a-5p, and let-7g-5p) were consistently highly expressed in the rumen tissues at the four developmental stages.

**Figure 1:**
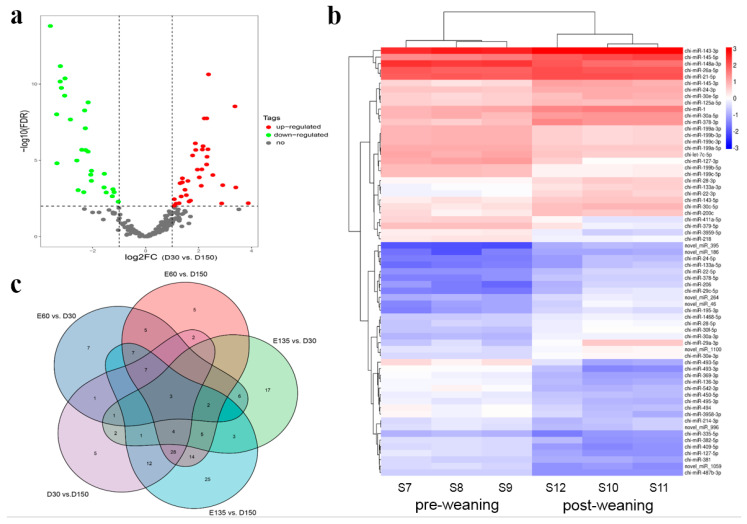
(**a**) Numbers of up-regulated and down-regulated differentially expressed miRNAs (DEMs) in goat rumens at D30 and D150. (**b**) Hierarchical clustering analysis of DEMs. The column indicates different samples. The row indicates DEMs. Red color indicates the highly expressed DEMs, and green color for the lowly expressed DEMs. (**c**) Venn diagram showing the common and unique DEMs in different pairwise comparisons.

**Figure 2:**
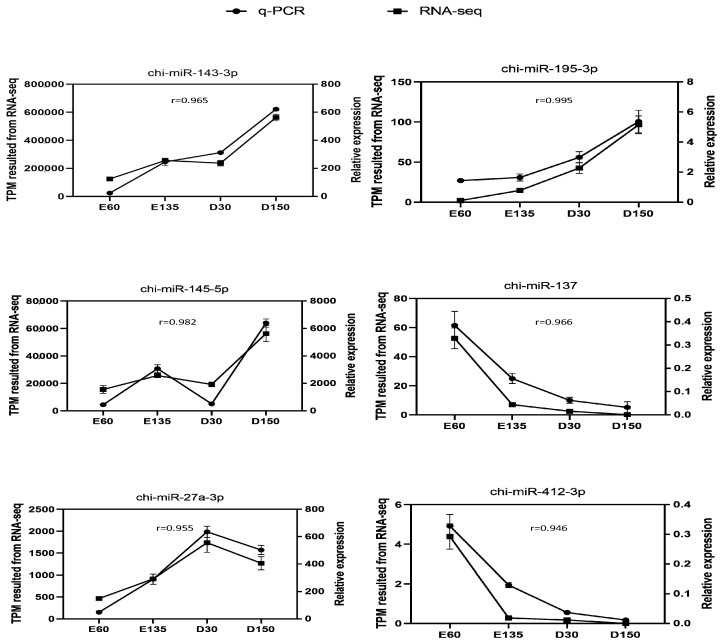
Validation of the six selected DEMs by qPCR.

In the post-natal period, 35 up-regulated DEMs and 31 down-regulated DEMs were observed between the rumens at D30 and D150 (Figure 1a, Supplementary Table S4). The hierarchical cluster of these 66 DEMs was shown in Figure 1b, indicating a consistent expression tendency for each stage. Interestingly, there were comparable DEMs in the pairwise comparison of E60 and D30 (27 up-regulated and 12 down-regulated) and the comparison of E135 and D30 (19 up-regulated and 25 down-regulated), while in the pairwise comparison of E60 and D150 (53 up-regulated and 29 down-regulated) and the comparison of E135 and D150 (67 up-regulated and 44 down-regulated), the number of DEMs was markedly increased (Supplementary Figure S2). The Venn diagram (Figure 1c) shows the DEMs in each comparison of two different developmental stages. The expression patterns of the selected DEMs were verified by qPCR and consistent with the tendency of the small RNA-sequencing data (Figure 2).

### 3.2. GO and KEGG Pathway Analyses of Putative Target Genes

**Figure 3:**
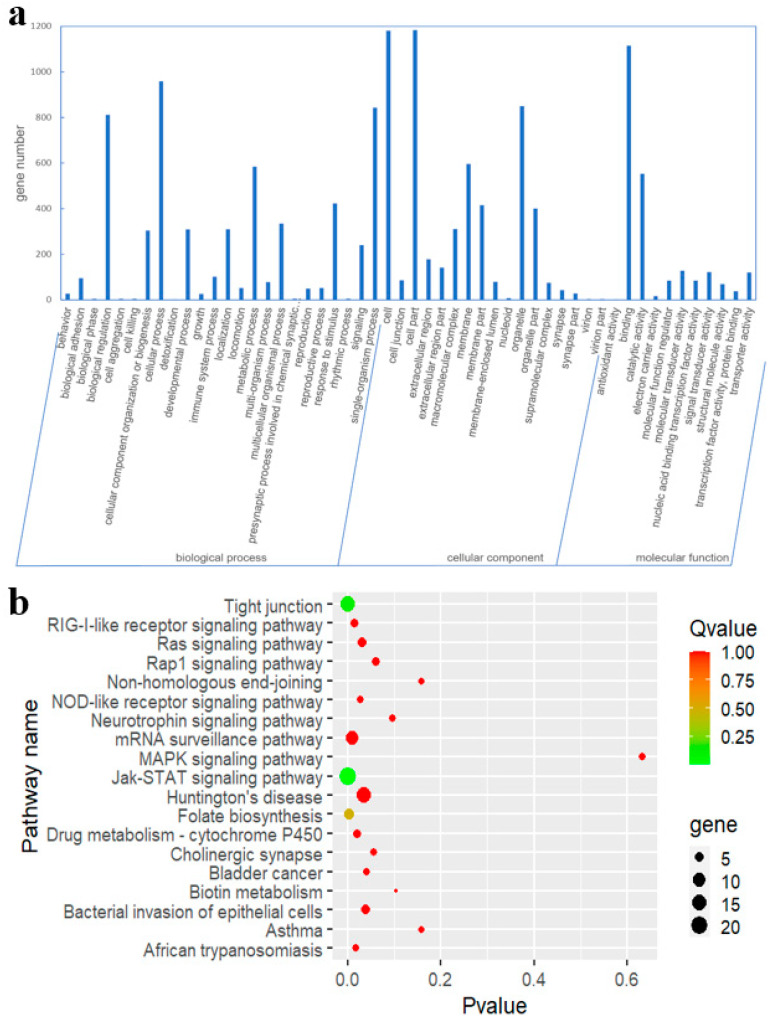
(**a**) GO functional classification of predicated target genes of the DEMs. (**b**) Enrichment analysis of KEGG pathway in target genes of the DEMs.

To elucidate the biological roles on development of rumens, a total number of 4622 target genes of the 166 DEMs were subjected to Gene Ontology (GO) analysis. Fifty-two GO terms were identified, including 24 biological processes, 17 cellular components, and 11 molecular functions (Figure 3a). The analysis of biological process showed that most genes were involved in cellular process and biological regulation. On the molecular function level, genes were mainly involved in binding and catalytic activity. On the cellular component level, genes were mainly related to organelle and cell parts. Meanwhile, the biological pathways involved in rumen development were revealed by the Kyoto Encyclopedia of Genes and Genomes (KEGG) analysis based on all the DEMs. In total, 188 signaling pathways were considered as significantly enriched (Supplementary Table S5), and involved in MAPK signaling pathway, Rap1 signaling pathway, Ras signaling pathway, and Jak-STAT signaling pathway, which were linked to cell proliferation (Figure 3b). In addition, six specific GO terms and thirty-five KEGG categories were revealed between embryonic and post-weaning stages (Supplementary Table S6). Furthermore, eight KEGG pathways were identified between pre-weaning and post-weaning periods, which mainly enriched in tryptophan metabolism and glycosaminoglycan biosynthesis (Supplementary Table S6).

### 3.3. Candidate miR-148a-3p Expression and Target Gene Analysis

**Figure 4:**
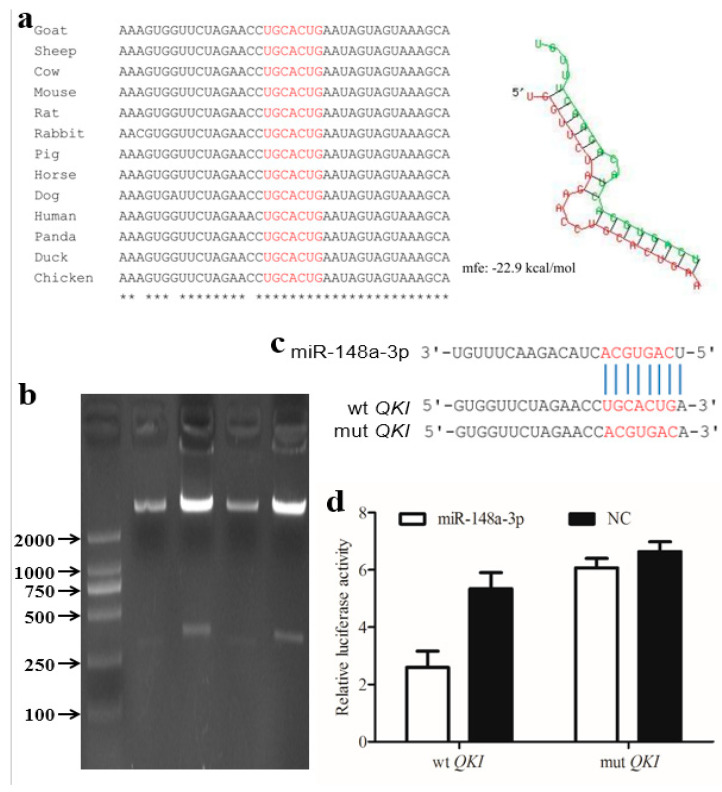
(**a**) Alignment of binding sites among different species and the predicted secondary structure of miR-148a-3p and targeting *QKI*. (**b**) Electrophoresis of the recombinant plasmid. The recombinant plasmid was approximate 6.4 KB. (**c**) Base complementarity between miR-148a-3p and the 3′-UTR of *QKI*. (**d**) Luciferase reporter assay of HEK293T cells co-transfected with *QKI* wild-type or *QKI* mutant-type and miR-148a-3p mimics.

The expression pattern of miR-148a-3p in the rumens is shown in Table 1, representing a high expression level in pre-weaning stages (E60, E135, and D30) and a low expression in the rumen at post-weaning period (D150). Thus, we proposed that miR-148a-3p may play an important role in rumen epithelium development. The potential target genes (Supplementary Table S7) of miR-148a-3p were estimated by the Targetscan analysis. Because correlation of expression of miR-148a-3p and *QKI* was high, *QKI* was selected as the target gene to be further investigated. The binding sites of *QKI* 3′-UTR are highly conserved in various species, including goat, sheep, and cow. The predicted minimum free energy of miR-148a-3p and *QKI* mRNA was found to be −22.9 kcal/mol (Figure 4a). The recombinant plasmid was successfully constructed using a length of 257 bp fragment from the 3′-UTR of *QKI* and the psiCHECK-2 vector (Figure 4b,c). The luciferase reporter activity of co-transfected miR-148a-3p mimics and wild type was significantly lower than that of co-transfected miR-148a-3p negative control and wild type (Figure 4d, *p* < 0.01). The luciferase reporter activity of co-transfected miR-148a-3p mimics and mutant was not significantly different from that of co-transfected miR-148a-3p negative control and mutant. In addition, the luciferase reporter activity of co-transfected miR-148a-3p mimics and wild-type mimics was significantly lower than that of co-transfected miR-148a-3p mimics and mutant mimics (*p* < 0.01). These results indicated that miR-148a-3p could bind to the 3′-UTR of *QKI*, thereby inhibiting the expression of *QKI*.

### 3.4. Co-Localization of miR-148a-3p and QKI in Goat Digestive Tissues

**Figure 5:**
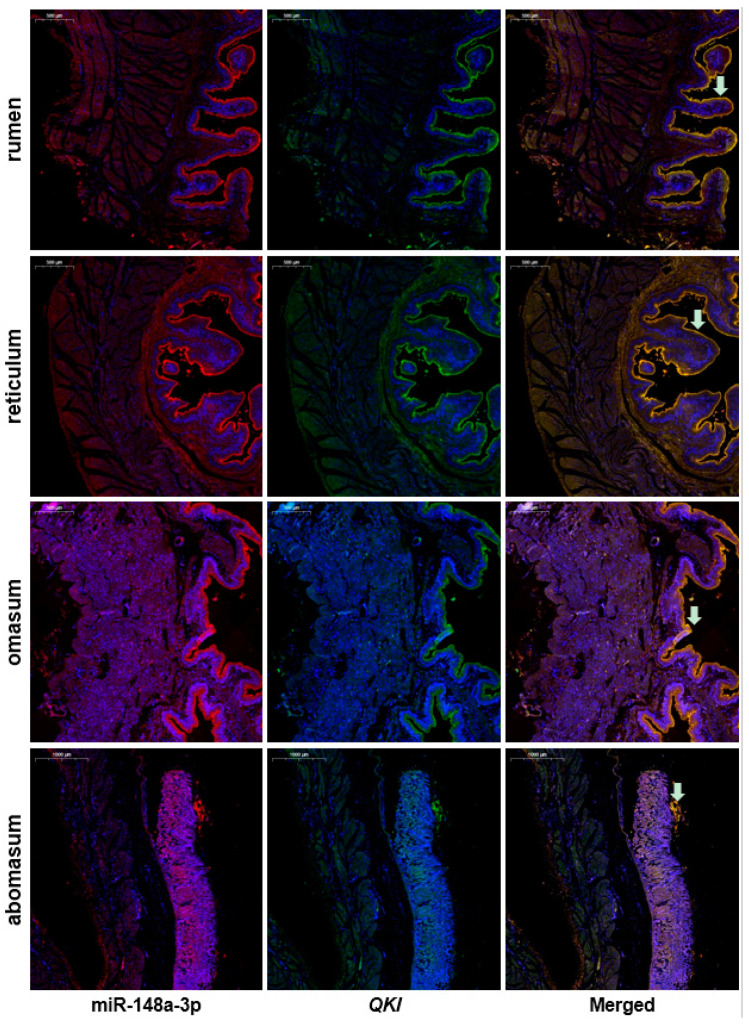
Co-location of miR-148a-3p and *QKI* in different stomach chambers. Bar. 500 μm. Arrow indicates the epithelial layer.

The co-localization of miR-148a-3p and *QKI* in rumen, reticulum, omasum, and abomasum was performed by double-color fluorescence in situ hybridization (Figure 5). The signal locations of miR-148a-3p and *QKI* were consistent, and miR-148a-3p and *QKI* were expressed in all layers of the gastric wall, but significantly higher in the epithelial layer than in other layers. To characterize the potential function of miR-148a-3p and *QKI* in the intestinal tract, we also investigated their co-localization in the duodenum, jejunum, ileum, and colon (Supplementary Figure S3). The results showed that miR-148a-3p and *QKI* were inconsistently expressed in many structures, and the expression levels of miR-148a-3p and *QKI* in different portions of intestinal tract were relatively low. In duodenal sections, miR-148a-3p was higher in the muscularis than in the villus, while *QKI* was highly expressed in the villus than that in the muscularis. The signal intensity of miR-148a-3p and *QKI* on the vascular wall of the colon was significantly higher than that of other structures. Co-localization of MiR-148 and *QKI* was restricted to the epithelium wall and, therefore, may be involved in its development or function.

### 3.5. miR-148a-3p Affects Cell Proliferation by Targeting QKI

**Figure 6:**
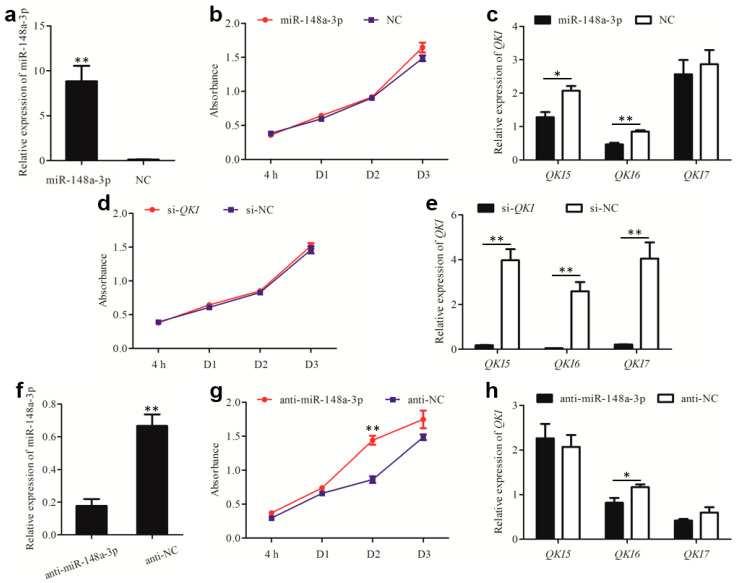
miR-148a-3p regulates cell proliferation of GES-1. (**a**) The efficiency of miR-148a-3p mimics transfection. (**b**) Growth curve of GES-1 cells transfected with miR-148a-3p mimics. (**c**) Relative expression of main isoforms of *QKI* after transfection with miR-148a-3p mimics. (**d**) Growth curve of GES-1 cells transfected with *QKI* siRNA. (**e**) The efficiency of *QKI* siRNA transfection. (**f**) The efficiency of miR-148a-3p inhibitor transfection. (**g**) Growth curve of GES-1 cells transfected with miR-148a-3p inhibitor. (**h**) Relative expression of main isoforms of *QKI* after transfection with miR-148a-3p mimics. * *p* < 0.05; ** *p* < 0.01.

To investigate the effect of miR-148a-3p on cell proliferation, we measured the cell count of GES-1 after co-transfection. As shown in Figure 6, expression of miR-148a-3p was determined by qRT-PCR after transfection, miR-148a-3p expression was significantly higher than that of the negative control (Figure 6a). Proliferation of GES-1 cells was then assessed at 4, 24 (D1), 48 (D2) and 72 h (D3) by the CCK8 kit; there was no significant difference in cell proliferation between the GES-1 cells overexpressing miR-148a-3p and the negative control (Figure 6b). As shown in Figure 6c, the expression levels of *QKI5* and *QKI6* in the GES-1 cells transfected by miR-148a-3p mimics were significantly down-regulated. Although there was no significant effect on the proliferation of GES-1 cells (Figure 6d), expression of *QKI5*, *QKI6* and *QKI7* was significantly down-regulated in GES-1 cells transfected with *QKI* siRNA (Figure 6e). Intriguingly, the expression of miR-148a-3p was significantly decreased after transfection with miR-148a-3p inhibitor (Figure 6f). As shown in Figure 6g, the proliferation of GES-1 cells was significantly increased after 48 h transfection. Compared to negative control, expression levels of *QKI5* were slightly up-regulated, while the expressions of *QKI6* were significantly down-regulated (Figure 6h). These data suggest that miR-148a-3p could inhibit the expression of *QKI5* by binding to its 3′-UTR. Furthermore, inhibition of miR-148a-3p expression could promote the proliferation ability of GES-1 cells.

## 4. Discussion

In this study, the expression characteristics of genome-wide microRNAs involved in rumen development and their potential influences on the functional transition of goat rumen epidermis were investigated. Seven highly expressed miRNAs, which are mainly involved in cell proliferation, growth and apoptosis, were identified at the four developmental periods. Among these, miR-21-5p and miR-26a-5p have also been shown to be associated with the proliferation of melanoma and hepatocellular carcinoma cells, respectively [27,28]. As for the members of let-7, expressions of let-7i-5p, let-7f-5p and let-7g-5p have been revealed to regulate the regeneration rate of liver in mice [29]. In domestic animals, miR-148a-3p was highly expressed in fetal bovine skeletal muscle, but decreased in the growing myocytes, indicating that the overexpression of miR-148a-3p could inhibit the proliferation of myocytes [18]. In rabbits, miR-148a-3p is not only highly expressed in white adipose tissue at early growth stage but also gradually increased in the differentiation process of preadipocytes cultured in vitro [30]. In pigs, miR-148a-3p was always highly expressed during the development of skeletal muscle, which was speculated to be related to the proliferation, generation and apoptosis of muscle cells [31]. In the current study, we found that miR-148a-3p was highly expressed in the D30 rumen and showed down-regulated expression at post-weaning period (D150). Compared with location in the rumen, miR-148a-3p and *QKI* were not expressed in the same location of the intestinal tracts (Supplementary Figure S3), indicating that miR-148a-3p may play a role in rumen development though *QKI* in a tissue-specific manner. Furthermore, miR-148a-3p suppressed the cell proliferation of GES-1 cells by regulating *QKI*, suggesting that a similarity has effects on the rumen epithelial cells. Thus, we proposed that miR-148a-3p could play an especially important role in the development of the rumen epithelium cells.

Several differential expressed genes (DEGs) have been identified in cattle rumen between pre- and post-weaning [2,32,33]. During weaning, the DEGs in calf rumen epithelium were mainly involved in the processes of lipid metabolism, cell morphology, growth and proliferation and molecular transport [2]. In addition, more DEGs (4104) were revealed between the pre- and the post-weaning periods in bovine rumen and several highly DEGs with immune functions (*LY6D*, *MUC1*, *EMB* and *EYA2*) were pointed out [32]. Similarity, immunity and lipid metabolism were also significantly enriched for DEGs in the rumen [32]. Furthermore, 122 DEMs were identified from 260 known and 35 novel miRNAs in the same rumen tissues. Among them, six miRNAs (miR-143, miR-29b, miR-145, miR-493, miR-26a and miR-199) were identified as the key regulators of rumen development in cattle [33]. From fetal to adulthood, the morphology, structure and function of the rumen change significantly, especially the surface area of the rumen papillae of goats under supplementary feeding significantly increased during the pre- and post-weaning periods [34]. Compared with the rumens at post-weaning period, a similar number of DEMs were detected in the pre-natal rumens (82 DEMs in the comparison of E60 and D150; 111 DEMs in the comparison of E135 and D150; Supplementary Figure S2). The target genes of these DEMs were mainly enriched in the cell cycle, except for the Rap1 signaling pathway and PI3K-Akt signaling pathway, indicating the different pathways involved in the embryonic development. However, the functional studies about these miRNAs in goat rumens are still limited.

To date, several investigations have focused on the molecular mechanism of rumen epithelial cell proliferation and related transporter regulatory pathways, such as insulin-like growth factor (IGF) and epidermal growth factor (EGF) involved in the regulation of glucose transport, NHE (sodium/hydrogen antiporter), MCTs (monocarboxylate cotransporter) and GPR involved in the transport of short-chain fatty acids (SCFA) in rumen epithelial cells [35,36]. In this study, the target genes of DEMs identified in goat rumens were significantly enriched in the MAPK signaling pathway, Jak-STAT signaling pathway, Rap1 signaling pathway, Ras signaling pathway and PI3K-Akt signaling pathway. Several factors could affect cell proliferation and differentiation mediated by the epidermal growth factor receptor (EGFR) in response to EGF and transmembrane transforming growth factor (TGF) through the MAPK signaling pathway [37], it promoted the growth of gastric epithelium during development and the renewal of the gastric tissue [38]. It has been shown that inhibition of the Jak-STAT3 signaling pathway could inhibit cell proliferation in gastric cancer cells [39]. Ras signaling was a downstream pathway of EGFR and played an important role in the cell fate and proliferation of intestinal epithelial cells [40]. In addition, phosphorylation of the PI3K-Akt signaling pathway can promote ghrelin-mediated intestinal cell proliferation [41]. Compared with pre-natal rumens (E60 and E135), the differently enriched pathways at the post-weaning stage were mainly clustered into carbohydrate digestion and absorption, vitamin digestion and absorption, adherens junction, gap junction and amino acid metabolism (Supplementary Table S5). Subsequently, the specific pathways including tryptophan and metabolism, ether lipid metabolism and glycosaminoglycan biosynthesis were revealed in comparison of D30 and D150. These results indicated that the pathways mainly involved in epithelial cell junctions, digestion and metabolism of nutrients during pre- and post-weaning periods.

## 5. Conclusions

In summary, the genome-wide expression profile of miRNAs and specific pathways involved in the development of the rumen were identified in goats. FISH and luciferase experiments verified that *QKI* was the target gene of miR-148a-3p. Furthermore, suppression of miR-148a-3p could induce the proliferation of GES-1 cells. Our results indicated that miR-148a-3p related to rumen development in goats by targeting *QKI*.

## Figures and Tables

**Table 1:** Information on high expression of miRNAs in the rumen of four different periods of goats.

miRNAs	Sequences (5′–3′)	E60 (TPM)			E135 (TPM)			D30 (TPM)			D150 (TPM)		
		S01	S02	S03	S04	S05	S06	S07	S08	S09	S10	S11	S12
chi-miR-143-3p	UGAGAUGAAGCACUGUAGCUCG	129,899	108,660	133,032	282,443	236,491	248,819	169,226	283,572	256,290	587,757	615,886	483,343
chi-miR-148a-3p	UCAGUGCACUACAGAACUUUGU	49,694.4	63,517.7	61,760	53,322.6	57,020.7	73,458.6	162,467	123,649	134,682	20,601.2	16,858.1	41,078.9
chi-miR-21-5p	UAGCUUAUCAGACUGAUGUUGAC	34,325.5	42,267.3	40,249.6	36,373.6	38,252.6	35,896.3	52,697.9	67,017.6	55,942.2	55,781.8	50,200.3	56,630.2
chi-miR-26a-5p	UUCAAGUAAUCCAGGAUAGGCU	42,298.4	29,747.9	38,170.5	37,739	42,638.6	38,951.3	58,679.8	50,392.9	45,516.7	43,799.4	40,410.2	53,231.2
chi-let-7f-5p	UGAGGUAGUAGAUUGUAUAGUU	48,221.2	37,486.7	48,836.2	39,702	48,107.2	43,888	42,274.6	27,161.3	34,006.6	18,615.6	18,521.2	29,367
chi-let-7i-5p	UGAGGUAGUAGUUUGUGCUGUU	89,488.7	59,637.3	80,713.4	48,114	55,336	54,656.8	103,139	72,450	83,735.8	24,565	18,454.3	40,592.7
chi-let-7g-5p	UGAGGUAGUAGUUUGUACAGUU	39,837.1	27,269.6	33,591.3	25,213.5	27,818.7	25,772	39,747.4	26,331.8	30,419.7	18,546.2	16,769	25,938
chi-miR-10a-5p	UACCCUGUAGAUCCGAAUUUGU	39,578.2	56,845	25,375.6	46,804.6	49,822.8	49,492.8	16,458.1	18,478	18,093.6	14,346.4	8928.24	12,395.3
novel_miR_816	GUGAAAUGUUUAGGACCACUAG	7716.68	10,842.2	5830.39	43,031.7	51,744.6	38,203.8	25,770.8	31,988.9	23,516	7712.28	6032.93	10,368.6
chi-miR-27b-3p	UUCACAGUGGCUAAGUUCUGC	22,255.2	22,535.4	26,817.6	34,768.4	35,165	32,998.9	22,256.4	24,188	22,918.6	18,671	19812.2	17,478.8
chi-miR-7-5p	UGGAAGACUAGUGAUUUUGUUGUU	40,591.3	12,547.5	37,260.2	2969.46	3014.75	3654.06	5162.28	3850.75	2571.84	1782.93	1498.76	2180.65
chi-miR-127-3p	UCGGAUCCGUCUGAGCUUGG	22,241.9	33,430.2	22,459.3	10,290.6	11,178.6	11,424.1	6808.42	9133.77	10,262.4	266.207	233.613	1682.39
chi-miR-99a-5p	AACCCGUAGAUCCGAUCUUGU	21,043.9	23,685.3	22,250.8	21,408.8	21,530.7	23,416.2	29,877.8	23,867	25,090.2	7432.25	6398.27	13,073.1
chi-miR-1	UGGAAUGUAAAGAAGUAUGUAU	6661.52	24,793.4	3678.91	12,652.4	12,841.1	10,218.5	5476.6	6946.71	8299.96	13,206.9	12,632.8	11,555.9
chi-miR-145-5p	GUCCAGUUUUCCCAGGAAUCCCU	23,695.1	6810.14	16,205.4	27,621.6	26,220.7	23,251.9	14,500.2	18,636.5	24,478.5	58,611.7	72,595	37,617.3

TPM: Transcripts Per Million.

## Supplementary Materials

The following are available online at https://www.mdpi.com/2076-2615/10/11/1951/s1, Table S1: Information about the primers and probes used in this experiment. Table S2: Summary of reads from raw data and clean reads for miRNAs sequencing in goat rumen. Table S3: List of the known-miRNAs and novel-miRNAs identified in this study. Table S4: The identified DEMs in goat rumens (D30 vs. D150). Table S5: The 188 KEGG pathway analysis of the DEMs target genes. Table S6: List of the unique GO and KEGG pathways of the DEMs target genes. Table S7: Candidate target genes for miR-148a-3p in goat rumens. Figure S1: Gene structure of *QKI* and locations of the used probes and primers. Red, green, purple and brown arrows indicate locations of probes, and qPCR primers for *QKI5*, *QKI6* and *QKI7*, respectively. Figure S2: Volcano plot of differentially expressed miRNAs in E60 vs. D30, E60 vs. D150, E135 vs. D30 and E135 vs. D150. The red and green dots represent up-regulated and down-regulated DEMs, respectively. Figure S3: Co-location of miR-148a-3p and *QKI* in goat intestinal tract. Bar. 500 μm. Star, triangle and arrows indicate the muscularis, villus and vascular wall, respectively.
