# Supplementary material for: Comparison of MicroRNA Transcriptomes Reveals the Association between MiR-148a-3p Expression and Rumen Development in Goats
Source: Animals (Basel). 2020 Oct 23;10(11):1951. doi: 10.3390/ani10111951 (PMC7690783; doi:10.3390/ani10111951)
Supplement: Supplementary file 1 [file animals-10-01951-s001.zip › Figure S1.ppt]

## Slide 1
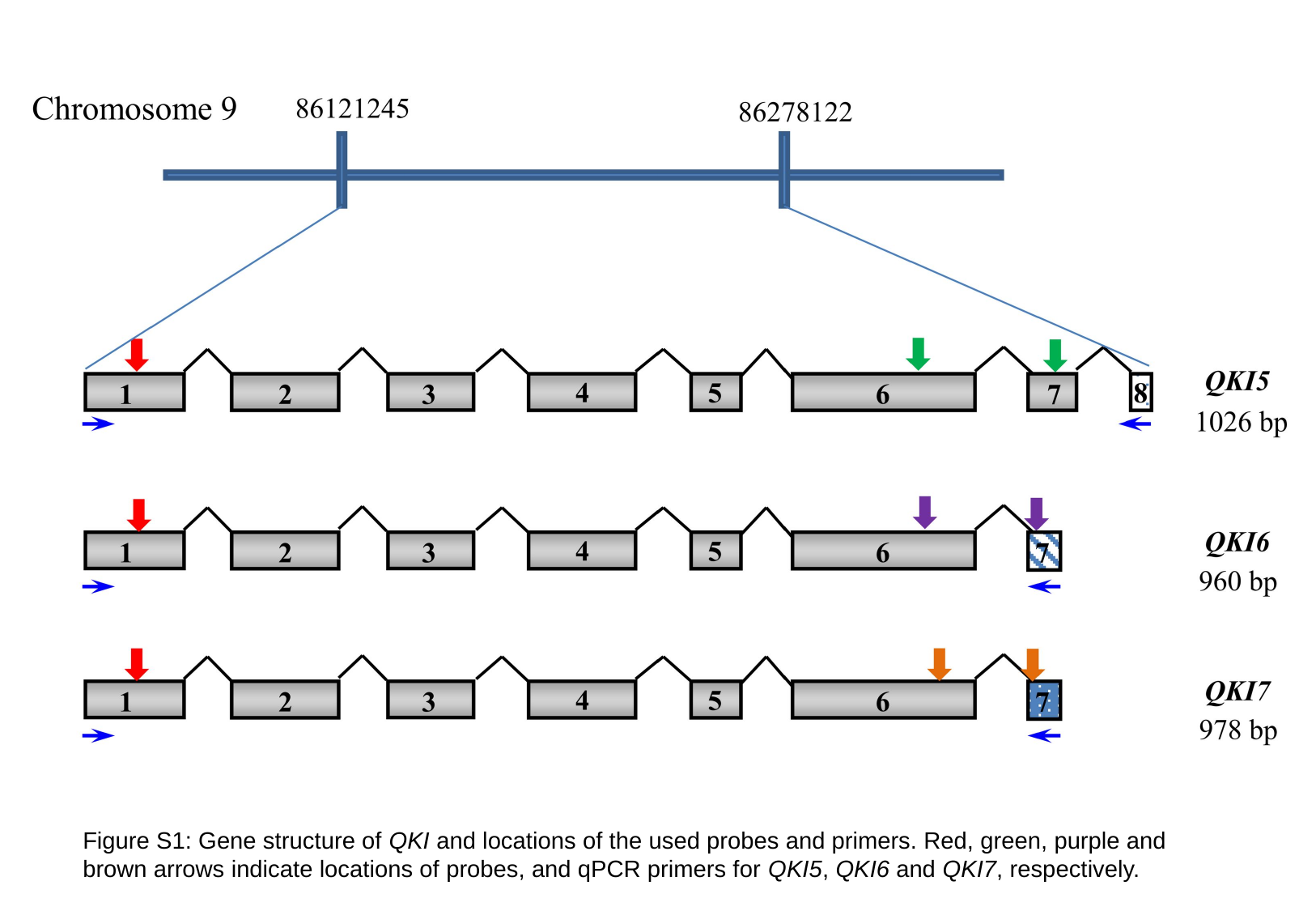

Figure S1: Gene structure of QKI and locations of the used probes and primers. Red, green, purple and brown arrows indicate locations of probes, and qPCR primers for QKI5, QKI6 and QKI7, respectively.
