# Supplementary material for: Comparison of MicroRNA Transcriptomes Reveals the Association between MiR-148a-3p Expression and Rumen Development in Goats
Source: Animals (Basel). 2020 Oct 23;10(11):1951. doi: 10.3390/ani10111951 (PMC7690783; doi:10.3390/ani10111951)
Supplement: Supplementary file 1 [file animals-10-01951-s001.zip › Table S2.doc]

**Table S2:** Summary of reads from raw data and clean reads for miRNAs sequencing in goat rumen.

| Term | E60 | | | E135 | | | D30 | | | D150 | | |
| --- | --- | --- | --- | --- | --- | --- | --- | --- | --- | --- | --- | --- |
|  | S01 | S02 | S03 | S04 | S05 | S06 | S07 | S08 | S09 | S10 | S11 | S12 |
| Raw reads | 19794964 | 18233301 | 18401922 | 18944864 | 21145362 | 23397815 | 20893095 | 20893095 | 23757258 | 20995225 | 22404153 | 19971937 |
| Unannotated reads | 14472895 | 11603309 | 15472475 | 16303990 | 18907593 | 18119774 | 17281703 | 15813553 | 22034942 | 18987088 | 20801315 | 17201358 |
| Mapped reads | 8095631 | 7329332 | 9793185 | 9478633 | 10630261 | 10931889 | 9531242 | 8243206 | 11705383 | 9063248 | 9267751 | 8315183 |
| Length<18 | 313616 | 797496 | 313920 | 357937 | 246494 | 601503 | 129722 | 120287 | 130770 | 68222 | 79240 | 143078 |
| Length>30 | 3426505 | 3589205 | 1463316 | 1121606 | 1100208 | 1100208 | 1763763 | 589766 | 788291 | 997311 | 738496 | 816982 |
| Clean reads | 16046608 | 13838938 | 16617249 | 17450284 | 19782216 | 20525735 | 18999610 | 16462197 | 22838197 | 19929692 | 21586417 | 19011877 |
| Q30 (%) | 92.61 | 92.05 | 92.93 | 93.85 | 93.85 | 93.85 | 98.63 | 98.72 | 98.73 | 98.49 | 98.66 | 98.67 |
