# Supplementary material for: Comparison of MicroRNA Transcriptomes Reveals the Association between MiR-148a-3p Expression and Rumen Development in Goats
Source: Animals (Basel). 2020 Oct 23;10(11):1951. doi: 10.3390/ani10111951 (PMC7690783; doi:10.3390/ani10111951)
Supplement: Supplementary file 1 [file animals-10-01951-s001.zip › Table S6.doc]

**Table S6:** List of the unique GO and KEGG pathways of the DEMs target genes.

| **Period** | **GO terms** | **GO id** |  |  |  |
| --- | --- | --- | --- | --- | --- |
| E vs. D150 | biological phase | GO0044848 |  |  |  |
|  | detoxification | GO0098754 |  |  |  |
|  | nucleoid | GO0009295 |  |  |  |
|  | virion | GO0019012 |  |  |  |
|  | virion part | GO0044423 |  |  |  |
|  | antioxidant activity | GO0016209 |  |  |  |
| **Period** | **Pathway** | **Ko id** | **count** | **P-value** | **Rich factor** |
| E vs. D150 | Cell cycle | ko04110 | 1 | 0.9653218 | 0.3184888 |
|  | p53 signaling pathway | ko04115 | 3 | 0.5017881 | 1.1374599 |
|  | Adherens junction | ko04520 | 2 | 0.3634315 | 1.5924439 |
|  | Gap junction | ko04540 | 2 | 0.6802089 | 0.8846911 |
|  | ErbB signaling pathway | ko04012 | 1 | 0.5531697 | 1.3270366 |
|  | ECM-receptor interaction | ko04512 | 2 | 0.2161571 | 2.2749199 |
|  | Protein export | ko03060 | 1 | 0.7389528 | 0.796222 |
|  | SNARE interactions in vesicular transport | ko04130 | 2 | 0.57892 | 1.0616293 |
|  | Non-homologous end-joining | ko03450 | 2 | 0.2161571 | 2.2749199 |
|  | Alanine, aspartate and glutamate metabolism | ko00250 | 1 | 0.800496 | 0.6635183 |
|  | Phenylalanine metabolism | ko00360 | 1 | 0.488936 | 1.5924439 |
|  | Phenylalanine, tyrosine and tryptophan biosynthesis | ko00400 | 1 | 0.3314823 | 2.6540732 |
|  | Valine, leucine and isoleucine degradation | ko00280 | 1 | 0.7014007 | 0.8846911 |
|  | Glycosaminoglycan degradation | ko00531 | 1 | 0.5531697 | 1.3270366 |
|  | Glycosphingolipid biosynthesis - lacto and neolacto series | ko00601 | 1 | 0.8667305 | 0.5308146 |
|  | Other glycan degradation | ko00511 | 2 | 0.3634315 | 1.5924439 |
|  | Glycerolipid metabolism | ko00561 | 1 | 0.7014007 | 0.8846911 |
|  | Biotin metabolism | ko00780 | 1 | 0.1255931 | 7.9622196 |
|  | Pantothenate and CoA biosynthesis | ko00770 | 1 | 0.7717872 | 0.7238381 |
|  | Glutathione metabolism | ko00480 | 1 | 0.8475431 | 0.56873 |
|  | beta-Alanine metabolism | ko00410 | 2 | 0.4106086 | 1.4476763 |
|  | Drug metabolism - cytochrome P450 | ko00982 | 4 | 0.0394399 | 2.8953526 |
|  | Osteoclast differentiation | ko04380 | 1 | 0.8667305 | 0.5308146 |
|  | Carbohydrate digestion and absorption | ko04973 | 1 | 0.800496 | 0.6635183 |
|  | Vitamin digestion and absorption | ko04977 | 1 | 0.8981712 | 0.4683659 |
|  | Progesterone-mediated oocyte maturation | ko04914 | 4 | 0.1818091 | 1.7693821 |
|  | Aldosterone-regulated sodium reabsorption | ko04960 | 2 | 0.4106086 | 1.4476763 |
|  | B cell receptor signaling pathway | ko04662 | 1 | 0.6093385 | 1.1374599 |
|  | NOD-like receptor signaling pathway | ko04621 | 3 | 0.0467928 | 3.4123798 |
|  | Dopaminergic synapse | ko04728 | 2 | 0.6151048 | 0.9952774 |
| D30 vs. D150 | Collecting duct acid secretion | ko04966 | 1 | 0.2139686 | 4.3230769 |
|  | Dorso-ventral axis formation | ko04320 | 1 | 0.3822235 | 2.1615385 |
|  | Ether lipid metabolism | ko00565 | 1 | 0.6479891 | 0.9976331 |
|  | Glycosaminoglycan biosynthesis-chondroitin sulfate/dermatan sulfate | ko00532 | 2 | 0.3238219 | 1.7292308 |
|  | Glycosaminoglycan biosynthesis-heparan sulfate/heparin | ko00534 | 1 | 0.8547353 | 0.5403846 |
|  | Mismatch repair | ko03430 | 1 | 0.3305683 | 2.5938462 |
|  | Pertussis | ko05133 | 1 | 0.4298999 | 1.8527473 |
|  | Tryptophan metabolism | ko00380 | 1 | 0.4739035 | 1.6211538 |
