# Supplementary material for: Comparison of MicroRNA Transcriptomes Reveals the Association between MiR-148a-3p Expression and Rumen Development in Goats
Source: Animals (Basel). 2020 Oct 23;10(11):1951. doi: 10.3390/ani10111951 (PMC7690783; doi:10.3390/ani10111951)
Supplement: Supplementary file 1 [file animals-10-01951-s001.zip › Table S7.doc]

**Table S7:** Candidate target genes for miR-148a-3p in goat rumens.

| Target Gene | Gene Description | Binding region | Correlation^*^ |
| --- | --- | --- | --- |
| *DYRK1A* | dual specificity tyrosine phosphorylation regulated kinase 1A | 3'-UTR, CDS | -0.246 |
| *RAB14* | RAB14 member RAS oncogene family | 3'-UTR | -0.686 |
| *RASSF8* | Ras association domain family member 8 | 3'-UTR, 5'-UTR | 0.804 |
| *DSTYK* | dual serine/threonine and tyrosine protein kinase | 3'-UTR | -0.645 |
| *PRNP* | prion protein | 3'-UTR | -0.104 |
| *MAP3K4* | mitogen-activated protein kinase kinase kinase 4 | 3'-UTR | -0.399 |
| *QKI* | QKI, KH domain containing RNA binding | 3'-UTR | 0.722 |
| *ALCAM* | activated leukocyte cell adhesion molecule | 3'-UTR | 0.240 |
| *TGIF2* | TGFB induced factor homeobox 2 | 3'-UTR | -0.198 |
| *FXR1* | FMR1 autosomal homolog 1 | 5'-UTR | 0.391 |

^*^ Correlation of the expressions of miR-148a-3p and target genes.
