# Supplementary material for: Comparison of MicroRNA Transcriptomes Reveals the Association between MiR-148a-3p Expression and Rumen Development in Goats
Source: Animals (Basel). 2020 Oct 23;10(11):1951. doi: 10.3390/ani10111951 (PMC7690783; doi:10.3390/ani10111951)
Supplement: Supplementary file 1 [file animals-10-01951-s001.zip › Table S1.doc]

**Table S1:** Information about the primers and probes used in this experiment.

| Primer name | Primer sequences (5′-3′) | Size (bp) | Tm (°C) |
| --- | --- | --- | --- |
| miR-137 | CGATTGCTTAAGAATACGCGTAGT |  | 57.0 |
| miR-143-3p | CGTGAGATGAAGCACTGTAGCTCG |  | 63.3 |
| miR-145-5p | GTCCAGTTTTCCCAGGAATCCCT |  | 63.3 |
| miR-195-3p | CCAATATTGGCTGTGCTGCTC |  | 57.0 |
| miR-148a-3p | TCAGTGCACTACAGAACTTTGT |  | 62.8 |
| miR-27a-3p | CGTTCACAGTGGCTAAGTTCCG |  | 55.7 |
| miR-412-3p | CGACTTCACCTGGTCCACTAGCT |  | 60.0 |
| miR-NC-202845 | CGAAACTCTGAATGAACTTTTTGG |  | 57.0 |
| miR-NC-372634 | CGCAGTTGTAGACTGAATTTCAAGT |  | 57.0 |
| miR-NC-827014 | CGCAAAAACCTGAGTGAACTTTCT |  | 59.0 |
| QKI5F | GACAGATACAGACCGCTG | 220 | 60.2 |
| QKI5R | TTGGTAAGGATGGACACG |  |  |
| QKI6F | CAGACAGATACAGACCGC | 187 | 57.1 |
| QKI6R | CGTTGGGAAAGCCATACC |  |  |
| QKI7F | CAAACGGAACTCCTCACC | 159 | 54.6 |
| QKI7R | GCATTTCAATCCACTCTA |  |  |
| ACTBF | CCTGCGGCATTCACGAAACTAC | 87 | 59.7 |
| ACTBR | ACAGCACCGTGTTGGCGTAGAG |  |  |
| Probe | Probe sequence (5′-3′) |  |  |
| miR-148a-3p | CY3-ACAAAGTTCTGTAGTGCACTGA |  |  |
| *QKI* | FAM(488)-GGCTTCGGCTTCTCCTTCGTTTCCAT |  |  |
